# Toward a Glutamate Hypothesis of Frontotemporal Dementia

**DOI:** 10.3389/fnins.2019.00304

**Published:** 2019-03-29

**Authors:** Alberto Benussi, Antonella Alberici, Emanuele Buratti, Roberta Ghidoni, Fabrizio Gardoni, Monica Di Luca, Alessandro Padovani, Barbara Borroni

**Affiliations:** ^1^Neurology Unit, Department of Clinical and Experimental Sciences, University of Brescia, Brescia, Italy; ^2^International Centre for Genetic Engineering and Biotechnology, ICGEB, Trieste, Italy; ^3^IRCCS Istituto Centro San Giovanni di Dio Fatebenefratelli, Brescia, Italy; ^4^Department of Pharmacological and Biomolecular Sciences, University of Milan, Milan, Italy

**Keywords:** frontotemporal dementia, frontotemporal lobar degeneration, glutamate, neurotransmitter, autoimmunity, transcranial magnetic stimulation

## Abstract

Frontotemporal dementia (FTD) is a heterogenous neurodegenerative disorder, characterized by diverse clinical presentations, neuropathological characteristics and underlying genetic causes. Emerging evidence has shown that FTD is characterized by a series of changes in several neurotransmitter systems, including serotonin, dopamine, GABA and, above all, glutamate. Indeed, several studies have now provided preclinical and clinical evidence that glutamate is key in the pathogenesis of FTD. Animal models of FTD have shown a selective hypofunction in *N*-methyl *D*-aspartate (NMDA) and α-amino-3-hydroxyl-5-methyl-4-isoxazolepropionic acid (AMPA) receptors, while in patients, glutamatergic pyramidal neurons are depleted in several areas, including the frontal and temporal cortices. Recently, a selective involvement of the AMPA GluA3 subunit has been observed in patients with autoimmune anti-GluA3 antibodies, which accounted for nearly 25% of FTD patients, leading to a decrease of the GluA3 subunit synaptic localization of the AMPA receptor and loss of dendritic spines. Other *in vivo* evidence of the involvement of the glutamatergic system in FTD derives from non-invasive brain stimulation studies using transcranial magnetic stimulation, in which specific stimulation protocols have indirectly identified a selective and prominent impairment in glutamatergic circuits in patients with both sporadic and genetic FTD. In view of limited disease modifying therapies to slow or revert disease progression in FTD, an important approach could consist in targeting the neurotransmitter deficits, similarly to what has been achieved in Parkinson’s disease with dopaminergic therapy or Alzheimer’s disease with cholinergic therapy. In this review, we summarize the current evidence concerning the involvement of the glutamatergic system in FTD, suggesting the development of new therapeutic strategies.

## Introduction

Frontotemporal dementia (FTD) is one of the most common neurodegenerative conditions after Alzheimer’s Disease (AD), characterized by behavioral abnormalities, language impairment, and deficits of executive functions (Bang et al., 2015). The different clinical features have been grouped in different variants, represented by the behavioral variant of FTD (bvFTD) (Rascovsky et al., 2011), the agrammatic variant of Primary Progressive Aphasia (avPPA) and the semantic variant of PPA (svPPA) (Gorno-Tempini et al., 2011). Over the past ten years, for a common sharing of the same genetic and pathological determinants, atypical extrapyramidal conditions, including Corticobasal Syndrome (CBS) and Progressive Supranuclear Palsy (PSP), but also motor neuron disease (MND), were grouped under the same frontotemporal lobar degeneration (FTLD) disease spectrum (Litvan et al., 1996; Armstrong et al., 2013; Burrell et al., 2016). Concomitantly, the structural and functional brain correlates of each phenotype have been precisely reported (Rohrer et al., 2011). FTLD selectively affects the frontal and temporal regions, in which the main neuropathological hallmarks are constituted primarily by tau or TAR DNA-binding protein 43 (TDP-43) depositions (Spillantini and Goedert, 2013; Neumann and Mackenzie, 2019).

The identification of genetic mutations associated with FTLD helped to elucidate the underlying pathology, with mutations in *Microtubule Associated Protein Tau (MAPT)* causing tau accumulation, and *Granulin (GRN)* or the expansion on *chromosome 9 open reading frame 72 (C9orf72)* being associated with TDP-43 inclusions (Borroni and Padovani, 2013). Lastly, reappraisal of the pathological criteria for subtyping FTLD cases has benefited from some refinements, being updated with recent immunohistochemical, biochemical, and genetic advances (Cairns et al., 2007). In addition to FTLD-Tau or FTLD-TDP, several other neuropathological depositions have been defined, including FTLD-FET [with positivity for the FET family of DNA/RNA-binding proteins, comprising the fused in sarcoma (FUS), TATA-binding protein-associated factor 2N (TAF-15) and Ewing sarcoma protein (EWS)], FTLD-UPS (with inclusions of proteins of the ubiquitin-proteasome system) and FTLD-ni (with no inclusions observed) (Sieben et al., 2012; Van Mossevelde et al., 2018). Other uncommon genetic mutations have been described, including *valosin containing protein* (*VCP*) (Watts et al., 2004; van der Zee et al., 2009), *sequestosome 1* (*SQSTM1*) (Rubino et al., 2012; Le Ber et al., 2013; van der Zee et al., 2014; Kovacs et al., 2016), and *TANK-binding kinase 1* (*TBK1*) (Freischmidt et al., 2015; Gijselinck et al., 2015; Pottier et al., 2015), with an underlying TDP-43 pathology, *charged multivesicular body protein 2B* (*CHMP2B*) (Skibinski et al., 2005; Holm et al., 2009), associated with FTLD-UPS, and *FUS* mutations (Broustal et al., 2010; Van Langenhove et al., 2010) probably associated to FTLD-FET (no autopsy confirmation in patients with FTD to date but only in patients with amyotrophic lateral sclerosis) (Benussi et al., 2015a).

Despite the giant step forward in the knowledge of clinical, imaging, genetic and biological underpinnings of the disease, the absence of a reliable biomarker to predict the ongoing neuropathology represents a major limit to develop disease-modifying therapies that target tau or TDP-43 deposits, and that could be administered only to subjects with known pathogenetic mutations (Bang et al., 2015; Borroni et al., 2015). Moreover, it is still unknown whether tau and TDP-43 deposits represent the initial mechanism or simply the result of other trigger events.

Indeed, two different approaches might be pursued in the next future for treatment purposes: on one hand, there is urgent need to develop diagnostic markers able to identify the specific proteinopathies associated with FTLD, on the other, it might be possible to characterize neurotransmitter deficits shared by the entire FTLD spectrum (Rohrer et al., 2011).

Emerging evidence has now shown that FTD is characterized by a series of changes in several neurotransmitter systems, including serotonin, dopamine, GABA and, above all, glutamate (Murley and Rowe, 2018) (see Table 1).

**Table 1:** Evidence for neurotransmitter deficits in frontotemporal dementia.

	Type of evidence			
Neurotransmitter	Neurobiological	Neurophysiological	Neuroradiological	Pharmacological
Glutamate	+	+	+	-
GABA	+	+	-	-
Serotonin	+	-	+	+
Dopamine	+	-	+	+
Acetylcholine	-	-	-	-
Noradrenaline	-	-	±	+

*+: evidence of neurotransmitter deficit; -: no evidence of neurotransmitter deficit*.

The recent identification of anti-AMPA GluA3 antibodies in the serum and in the cerebrospinal fluid (CSF) from FTLD patients (Borroni et al., 2017) has suggested that the impairment of glutamate neurotransmission through an autoimmune mechanism might be considered as a possible target to slow or revert the disease. In this framework, we can hypothesize that a restoration of the appropriate glutamatergic stimulation could be reached by modulating (i) the immune system or (ii) the glutamatergic receptors, developing the latter approach in analogy to what has been demonstrated effective for Parkinson or Alzheimer disease, with dopaminergic and cholinergic therapies, respectively (Murley and Rowe, 2018).

In this review, we summarize the current evidence concerning the involvement of the glutamatergic system in FTD, suggesting the development of new therapeutic strategies.

## Molecular Biology

Glutamate, which represents the main excitatory neurotransmitter in the brain, largely contributes to memory and learning processes (Bliss and Collingridge, 1993), while being also involved in brain damage when abnormally activated in several conditions, including brain ischemia, epilepsy and neurodegeneration (Bowie, 2008). Glutamate exerts its functions at the synaptic level through both ionotropic (iGluR) and metabotropic glutamate receptors (mGluR).

iGluR are cation permeable tetramers, distinguished in *N*-methyl *D*-aspartate (NMDA), α-amino-3-hydroxyl-5-methyl-4-isoxazolepropionic acid (AMPA) and kainate (KA) on the basis of their affinity properties, the selectivity for different ions and the ability to generate rapid or slow electric kinetics. NMDA receptors (NMDAR) are known to mediate plasticity phenomena, as long-term potentiation (LTP) (Lescher et al., 2012), with a critical role of extra synaptic receptor subtype 2B (NR2B) subunit-containing ones (Paoletti et al., 2013). AMPA receptors are primarily involved in synaptic plasticity by modifications of subunits editing and composition, or interactions with different receptors and phosphorylation (Opazo et al., 2010; Huganir and Nicoll, 2013).

mGluR are a family of receptors coupled to G proteins, activating different transduction signals, mainly represented by phospholipase C and adenylate cyclase (Castillo et al., 2010). mGluRs contribute to neuronal plasticity and cognitive abilities, being able to mediate self-dependent forms of LTP and long-term depression (LTD) (Wang et al., 2015; Longhena et al., 2017).

Several evidences arise from both preclinical and clinical studies, showing the involvement of the glutamate neurotransmitter receptors, both iGluR and mGluR in the pathogenesis of FTLD.

In murine cortical neurons, silencing the FTD-associated gene granulin (*GRN*) decreases the expression of extra synaptic NR2B-containing NMDAR (Longhena et al., 2017); on the other hand, hyper-phosphorylated tau enhances glutamate release and produces an overactivation of the same receptor ending with neuron death, that can eventually be reduced by stimulating its reuptake through the astrocytic glutamate transporter 1 (GLT1)/excitatory amino acid transporter 2 (EAAT2) (Decker et al., 2016). Furthermore, FTLD has been related to the dysfunction in RNA pathways (Sephton and Yu, 2015), as corroborated by evidence that *FUS* depletion downregulates the transcription of GluA1, an essential AMPA-subunit involved in LTP phenomena (Udagawa et al., 2015). In that regard, also *charged multivesicular body protein 2b (CHMP2B)* FTD-related mutation increases GluA2 expression by disrupting microRNA levels (Gascon et al., 2014).

Knock out of the *glutamate ionotropic receptor AMPA type subunit 3* gene *(GRIA3)* produces modifications in social behavior with an increase in aggressiveness (Adamczyk et al., 2012): in a recent study GluA3-containing AMPAR turned to be dormant receptors, triggered by a peculiar intracellular signaling pathway (Renner et al., 2017). Neuronal activity stimulated by AMPAR activation induces tau release from mature cortical neurons in a calcium-dependent way, suggesting the glutamatergic modulation as a further approach to prevent tau depositions (Pooler et al., 2013). Autoantibodies for the GluA3 subunit of AMPARs have been identified both in the serum and CSF of FTD patients (Borroni et al., 2017), characterized by a bvFTD phenotype with presenile onset, absence of an autosomal dominant pattern of inheritance, and greater bitemporal atrophy. These anti-GluA3 antibodies lead to a reduction of the synaptic levels of GluA3-containing AMPARs both in rat primary neurons and in human neurons differentiated from induced pluripotent stem cells (iPSCs). In addition, the presence of GluA3 antibodies in the CSF induced a loss of dendritic spine density, and increased levels of tau protein *in vitro* human neurons (Borroni et al., 2017).

Interestingly, Leuzy et al. (2016) reported a reduced availability of mGlur5 in bvFTD patients. Several observations argued for a link between autoimmunity and FTD (Alberici et al., 2018), and more recently, it was demonstrated a significant increase in frequency of anti-nuclear antibodies (ANA) observed in FTD patients, as compared to normal control subjects (Cavazzana et al., 2018). According to these findings, it might be hypothesized that an immune system dysregulation results into an abnormal production of autoantibodies directed against the GluA3 subunit, causing a deficit in glutamatergic transmission, eventually leading to FTLD.

The involvement of glutamatergic transmission has also been reported in amyotrophic lateral sclerosis (ALS), which is part of the FTLD-ALS spectrum disorder, in which a glutamate-induced excitotoxicity of motor neurons has been hypothesized (Blasco et al., 2014). Deficient editing of the GluR2 AMPA receptor subunit (Kawahara et al., 2004) and a diminished functional transport of glutamate and reduced EAAT2 immunoreactivity has been observed in motor neurons of patients with ALS (Rothstein et al., 1992, 1995). These findings further support the possible complex role of glutamatergic transmission abnormalities in the pathophysiology of FTD-ALS.

Other possible modulators of glutamatergic transmission which have been shown to be impaired in FTD are serotonin (5-HT) and GABA. 5-HT has been shown to differently modify glutamate mediated effects, acting on distinct 5-HT receptor subtypes both at the pre-synaptic and post-synaptic site and in different brain regions: in the frontal cortex glutamate release is inhibited by serotonin whereas in the prefrontal cortex serotonin enhances glutamatergic transmission (Dawson et al., 2001; Ciranna, 2006). In FTD, a dysfunction of the serotoninergic system has been frequently observed (Bowen et al., 2008; Vermeiren et al., 2016), possibly opening an avenue for glutamatergic modulation through serotonin regulation (Huey et al., 2006).

Furthermore, GABA, which is the predominant inhibitory neurotransmitter in the brain with different functions other that merely counteracting excitatory glutamatergic neurons, has been shown to be impaired in FTD patients. Initial studies have shown that a subgroup of GABAergic neurons that bind calbinidin-D28k are reduced in the upper neocortical layers of the frontal and temporal cortices in FTD (Ferrer, 1999), while gamma oscillations and coherence, which reflect GABA inhibition, are reduced between the frontal lobes of patients with behavioral variant FTD (Hughes et al., 2018). These findings are corroborated by reports of the toxic effects mediated by tau and TDP-43 on GABAergic interneurons, leading to a loss of GABAergic function in animal models (Levenga et al., 2013; Yamashita and Kwak, 2014).

## Neurophysiology

Indirect evidence of the involvement of the glutamatergic system in FTD also comes from neurophysiological studies using both *in vitro* and *in vivo* techniques.

*In vitro* studies in transgenic mice expressing pathological human tau (V337M mutation), which is one of the main pathological hallmarks of FTD, have shown both AMPA and NMDA receptor hypofunction in the ventral striatum and insular cortex, which were reversible after the administration of cycloserine, an NMDA receptor co-agonist (Warmus et al., 2014). Further *in vitro* studies in transgenic mice carrying a *CHMP2B* mutation, which is another gene associated with FTD, have also shown altered AMPA receptor composition and function in the medial prefrontal cortex (Gascon et al., 2014).

*In vivo* neurophysiological evidence of the involvement of glutamatergic circuits in FTD mainly comes from non-invasive brain stimulation studies using transcranial magnetic stimulation (TMS) (Borroni et al., 2018). In this context, different paired-pulse TMS paradigms have been implemented to assess intracortical inhibitory and excitatory interneuronal circuits (Benussi et al., 2015b; Rossini et al., 2015). In particular, intracortical facilitation (ICF), which consists in a physiological facilitation elicited by applying a subthreshold conditioning magnetic stimulus followed by a suprathreshold test stimulus at an inter stimulus interval of 6–30 ms, has shown to depend mainly on glutamatergic circuits in the primary motor cortex (Ziemann et al., 2015), with NMDA receptor antagonists decreasing ICF (Ziemann et al., 1998; Schwenkreis et al., 1999).

Reduced ICF has been observed in patients with genetic FTD, carrying a *GRN* or *C9orf72* mutation, even in the presymptomatic phases of disease, compared to non-carrier first degree relatives (Benussi et al., 2016). These dysfunctions correlated with reduced cortical thickness and surface area of the right insula in presymptomatic *GRN* carriers, suggesting that glutamatergic impairment in the presymptomatic phases of *GRN*-related FTD could reflect the beginning of insular dysfunction, even in absence of cognitive or behavioral abnormalities (Gazzina et al., 2018).

Recently, the reduction of ICF has been observed up to 30 years before expected symptom onset in a very large cohort of *GRN* and *C9orf72* mutation carriers compared to non-carriers, long before the onset of clinical and neuroimaging abnormalities (Benussi et al., 2019).

An impairment of ICF has also been observed in sporadic FTD (Burrell et al., 2011; Benussi et al., 2017), confirming how this biomarker may be useful not only to track disease progression, but also to distinguish FTD from other forms of dementias, even in the early disease stages (Benussi et al., 2018a; Padovani et al., 2018).

Regarding other syndromes in the FTLD spectrum, a reduced ICF has been also observed in patients with CBS and PSP, highlighting how this technique may also be used to distinguish other atypical parkinsonian disorders, including dementia with Lewy bodies (Benussi et al., 2018b).

Alterations in ICF have been observed also in patients with both sporadic and familial ALS; however, contrary to what has been observed in FTD, an increase in ICF seems to be predominant (Geevasinga et al., 2015; Van den Bos et al., 2018). It is still debated if cortical hyperexcitability might act as an adaptive process in response to peripheral neurodegeneration and could serve as a neuroprotective strategy, or if cortical hyperexcitability may serve as a final common pathway in ALS, mediating neuronal degeneration via a trans-synaptic glutamate process (Geevasinga et al., 2015).

## Treatment Approaches: Targeting Glutamatergic Neurotransmission

Currently there are no approved treatments for FTD, and there are no therapies able to stop or alter the disease course. Pharmacological treatments to date have mostly concerned the off-label use of medications for symptomatic management. Recent advancements in understanding the molecular and genetic basis of FTD, and several clinical trials based on these insights are underway and have been reviewed elsewhere (Tsai and Boxer, 2016).

Glutamate neurotransmission has been considered a possible target for FTD symptomatic treatment. Memantine, a NMDA receptor antagonist with an indication for the treatment of moderate to severe AD (Tariot et al., 2004), was studied in two randomized, placebo-controlled trials over 52 and 26 weeks in FTD (Vercelletto et al., 2011; Boxer et al., 2013). Both studies failed to demonstrate significant benefits on behavioral disturbances or clinical global impression of change.

The recent observations of an effect exerted by the AMPARs activation on tau aggregation renewed the interest of glutamatergic modulation as a further approach to prevent tau depositions (Pooler et al., 2013; Borroni et al., 2017). Moreover, the identification of autoantibodies directed against GluA3 subunits provided evidence for an autoimmune dysregulation as a possible pathogenetic mechanism in FTD (Alberici et al., 2018). The link between autoimmune antibodies and neurodegeneration has been previously shown in the anti-IgLON5-related tauopathy, in which extensive neuropathological tau and TDP-43 inclusions have been observed (Sabater et al., 2014), placing these disorders at the convergence of neurodegenerative and autoimmune mechanisms. However, further research is necessary to validate these findings and elucidate the mechanisms by which these, or still other unidentified auto-antibodies, induce pathologic protein aggregates and neurodegeneration.

The feasibility of targeting an autoimmune response is an attractive potential therapeutic approach, suggesting immunomodulatory therapies as an evidence-based approach to treat FTLD. In the absence of prospective and randomized clinical trials for the treatment of autoimmune encephalitis, literature data are based on case reports with anti-NMDA, or more rarely, anti-AMPA receptor encephalitis (Dalmau and Graus, 2018). We can hypothesize that scavenging anti-GluA3 antibodies by using immunomodulation might restore glutamatergic transmission, thus slowing or reverting FTLD neurodegenerative process. Alternatively, in agreement with the glutamatergic hypothesis, and in analogy to what has been proposed for schizophrenia, positive allosteric modulators of AMPA receptors as well as orthosteric ligands and modulators of metabotropic glutamatergic receptors in particular ligands acting on mGlu receptors might be considered promising potential medications in FTLD (Menniti et al., 2013).

The modulation of glutamatergic transmission via 5-HT regulation may also be a promising approach to seek. Favorable evidence with selective serotonin reuptake inhibitors (SSRIs) has been observed in FTD patients, with several open label and placebo-controlled studies with SSRIs showing an improvement of several behavioral symptoms, as disinhibition, irritability and depression (Moretti et al., 2003; Lebert et al., 2004; Anneser et al., 2007; Herrmann et al., 2012; Hughes et al., 2015). However, it is still not known if this is a direct effect on serotoninergic transmission or possibly an indirect downstream effect on glutamatergic systems.

Regarding GABAergic therapies, evidence is currently lacking for a clinical efficacy in FTD patients.

## Conclusion

We have observed how the involvement of the glutamatergic system may play a key role in the pathogenesis of FTD both from a biological and neurophysiological perspective. This implication may open several avenues regarding treatment options which will have to be verified experimentally, both from a symptomatic but also possibly disease modifying approach.

**Figure 1:**
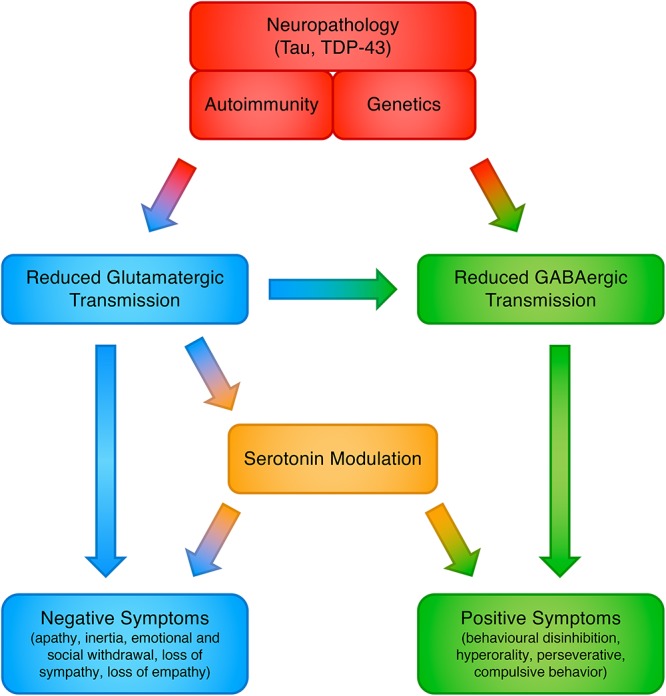
Proposed model for the involvement of neurotransmitter systems in frontotemporal dementia.

The involvement of glutamate in FTLD may answer some of the open issues in this field, yet we caution that FTD symptoms almost certainly do not flow from a single neurotransmitter abnormality. Indeed, this proposed model does not negate the involvement of other neurotransmitters, which have already been observed in FTLD, including GABA, serotonin and dopamine, and all of these may be ultimately brought together in a unified and interconnected framework (Murley and Rowe, 2018) (see Figure 1). Restoring these deficits, individually or in combination, has the potential to improve cognitive, behavioral and motor symptoms. More realistically, in fact the ultimate phenotypic expression probably arises from combinations of neurotransmitter abnormalities, genetic mutations, and environmental factors; combinations that may vary considerably from patient to patient.

Another interesting avenue worth pursuing is the potential for this amino-acid to act as a biomarker, either in establishing the diagnosis or as a measure of disease progression. Direct measurements in the CSF have shown a negative correlation between glutamate levels with verbally agitated behavior in FTD patients (Vermeiren et al., 2013). On the other hand, indirect measurements come from magnetic resonance spectroscopy of FTD patients in which glutamate/glutamine levels have been found to be reduced in the frontal and temporal lobes (Ernst et al., 1997; Sarac et al., 2008) and from neurophysiological studies with TMS, showing in both sporadic and genetic FTD a reduced ICF, which is partially mediated by glutamatergic transmission. In future, glutamate levels could also be indirectly assessed with electroencephalography (EEG) (Lally et al., 2014) or by TMS-EEG evoked potentials (Cash et al., 2016). To define which direct or indirect biomarker of glutamatergic neurotransmission might be the most useful and informative has still to be elucidated, considering the lack of studies on the subject, with different biomarkers perhaps providing distinct information from both a physiopathological and topographical perspective.

In conclusion, it is therefore now clear that the role of glutamate in FTD can represent an interesting and innovative approach to better understand the underlying ongoing neurodegenerative process in this pathology, although further investigations will be needed in order to increase our biological understanding of the disease, which will probably be contingent to the development of appropriate models and biomarkers for glutamatergic drug development.

## Author Contributions

All authors gave their substantial contribution to conception and design of the manuscript and drafting the manuscript and revising it critically for important intellectual content, approved the manuscript in its present form for publication, and agreed to be accountable for all aspects of the work in ensuring that questions related to the accuracy or integrity of any part of the work are appropriately investigated and resolved.

## Conflict of Interest Statement

The authors declare that the research was conducted in the absence of any commercial or financial relationships that could be construed as a potential conflict of interest.
